# Efficacy of Teduglutide in Pediatric Short Bowel Syndrome: Association with Citrulline Levels and Anatomical Location of Remnant Small Intestine

**DOI:** 10.3390/children12080977

**Published:** 2025-07-24

**Authors:** Yudai Goto, Kouji Masumoto, Takato Sasaki, Kazuki Shirane, Tomohiro Aoyama, Naoya Sakamoto, Takahiro Jimbo

**Affiliations:** Department of Pediatric Surgery, Faculty of Medicine, University of Tsukuba, Tsukuba 3058575, Ibaraki, Japan; kmasu@md.tsukuba.ac.jp (K.M.); ta-sasaki@md.tsukuba.ac.jp (T.S.); shirane.kazuki.nh@ms.hosp.tsukuba.ac.jp (K.S.); aoyama.tomohiro.ol@ms.hosp.tsukuba.ac.jp (T.A.); nsakamoto.pdsrg@md.tsukuba.ac.jp (N.S.); jimtaka@md.tsukuba.ac.jp (T.J.)

**Keywords:** short bowel syndrome, citrulline, GLP-2 analogue, teduglutide, children

## Abstract

**Highlights:**

**What are the main findings?**

- Teduglutide treatment increased plasma citrulline levels and reduced parenteral nutrition (PN) dependence in all four pediatric patients with short bowel syndrome (SBS), regardless of bowel anatomy.
- The degree of response in citrulline levels and PN reduction varied by SBS type, which suggest a potential influence of residual bowel anatomy on treatment outcomes.

**What are the implications of the main findings?**

- Plasma citrulline may serve as a useful biomarker for monitoring teduglutide efficacy, but its interpretation should consider individual bowel anatomy.
- Further studies are required to better understand the variable response to GLP-2 analogues and refine treatment evaluation in pediatric SBS.

**Abstract:**

**Background/Objectives:** Short bowel syndrome (SBS) is the leading cause of pediatric intestinal failure. Plasma citrulline is considered a marker indicating an enterocyte volume and may help evaluate the response to teduglutide; however, this interpretation may vary depending on the remnant bowel anatomy. **Methods:** We conducted a retrospective case series of four pediatric patients with SBS (aged < 15 years) who received teduglutide for 12 months at our hospital between 2018 and 2023. Changes in plasma citrulline levels and parenteral nutrition requirements were assessed in addition to bowel anatomy classification. **Results:** This study included two males and two females. All patients showed an increase in plasma citrulline levels and a reduction in the requirement for parenteral nutrition (PN) after 12 months of teduglutide treatment. In SBS type 2 (jejunocolic anastomosis), citrulline levels increased by 114% and 52%, with PN reduction rates of 100% and 30%, respectively. In SBS type 3 (jejunoileal anastomosis), citrulline levels increased by 13.6% and 34%, with PN reductions of 33% and 73%, respectively. **Conclusions:** Teduglutide treatment increased plasma citrulline levels and reduced PN levels in all cases. However, the magnitude of the citrulline change varied across bowel anatomy types, suggesting that the anatomical difference in the remnant bowel may influence the biomarker response. Further detailed pediatric cases are required to clarify the role of citrulline in evaluating GLP-2 analogue treatment outcomes.

## 1. Introduction

Short bowel syndrome (SBS) is the most common cause of intestinal failure in children. Patients with SBS exhibit impaired nutrient absorption and often require long-term parenteral nutrition (PN) to support their growth, hydration, and development. However, prolonged PN is associated with various complications, including intestinal failure-associated liver disease and catheter-related bloodstream infections, which can significantly reduce the child’s quality of life [1,2]. Therefore, the primary goal of treatment in pediatric SBS is to achieve enteral autonomy, which is defined as successful weaning from PN while maintaining appropriate growth and developmental outcomes.

Plasma citrulline is considered a useful biomarker indicating remnant enterocyte volume in patients with SBS [3,4]. This amino acid is found in only small amounts in the diet and is almost exclusively produced by enterocytes in the body [5]. In the absence of renal dysfunction, citrulline is converted to arginine in the kidneys, allowing its circulating levels to reflect the intestinal epithelial volume [6]. Although several studies have reported on plasma citrulline levels in patients with SBS, findings regarding its correlation with the remnant small bowel length, intestinal absorptive capacity, and PN requirements, have not yielded consistent results. Some reports have described negative correlations between citrulline levels and PN volume, whereas others have failed to confirm such an association [4]. Proli recently suggested that repeated citrulline measurements, when interpreted in the context of the SBS anatomical type, may serve as a reliable marker for subsequent PN weaning [7].

Teduglutide (REVESTIVE^®^, Takeda Pharmaceutical Company Limited, Tokyo, Japan), a glucagon-like peptide-2 (GLP-2) analogue, is the first effective therapy approved for the treatment of patients with SBS who are dependent on PN [8]. Jeppesen et al. reported that significant reductions in PN volume requirements were observed in adult patients with both a stoma and no colon-in-continuity and in those with high baseline PN volumes [4].

Recently, teduglutide has been shown to be effective in pediatric patients, with more than 80% of pediatric patients exhibiting a clinical response, including decreased PN fluid volumes and energy requirements [9,10,11]. In addition, teduglutide treatment has been associated with increased plasma citrulline levels compared to the baseline, which is considered to reflect pharmacodynamic effects on intestinal mass [10]. Wales et al. reported that colon-in-continuity was more strongly associated with successful weaning from PN support than the percentage of the remaining colon. They suggested that future research should examine whether the distal small intestine and right colon are preserved, as these regions are important sites for GLP-2 production [11].

This study evaluated changes in plasma citrulline levels in pediatric patients with SBS before and after 12 months of teduglutide administration. In addition, we investigated the relationships between citrulline levels, residual bowel anatomy and length, and the degree of dependence on PN.

## 2. Materials and Methods

### 2.1. Study Design and Setting

This retrospective study was conducted at a university hospital between 2018 and 2023. The aim was to evaluate the changes in plasma citrulline levels before and after 12 months of daily teduglutide administration in pediatric patients with SBS and to explore their relationship with residual bowel anatomy.

### 2.2. Participants

Eligible participants were pediatric patients aged <15 years diagnosed with SBS and receiving daily teduglutide treatment for 12 months. Teduglutide was initiated in patients whose PN requirements had stabilized following the intestinal adaptation period and for whom a further reduction in PN was considered difficult. Patients with renal failure, decompensated liver failure, or intestinal dysmotility were excluded. Four patients were included in this case series.

### 2.3. Exposure and Outcomes

Teduglutide was administered subcutaneously at a dose of 0.05 mg/kg/day, once daily. Abdominal and cardiac ultrasound evaluations were routinely performed before and during treatment. Given the challenges of gastrointestinal endoscopy in infants, contrast radiographic studies were used to exclude intestinal obstruction.

The exposure of interest was the 12-month administration of teduglutide. The primary outcome was the change in plasma citrulline levels from baseline (before teduglutide initiation) to 12 months post-treatment. Secondary observations included changes in the PN volume and energy intake.

### 2.4. Variables and Data Sources

Plasma citrulline samples were collected in the morning before oral intake and were measured by outsourced laboratory testing using liquid chromatography–tandem mass spectrometry (LSI Medience Corporation, Tokyo, Japan). Data on body weight, transthyretin levels, PN volume, and energy intake were obtained from electronic medical records. Body weight SD scores were calculated using reference values from the Infant Physical Growth Survey issued by the Japanese Ministry of Health, Labou, and Welfare. Information on residual bowel anatomy, including the presence or absence of the ileocecal valve and measurement of the remaining bowel length, was extracted from the surgical records. Classification of residual bowel anatomy was performed according to the anatomical classification system proposed by the European Society for Clinical Nutrition and Metabolism (ESPEN), which defines three types of SBS [12]: (1) SBS with end jejunostomy (type 1), resulting from the resection of both the ileum and colon; (2) SBS with jejuno–colic anastomosis (type 2), where most or all of the ileum is resected but part of the colon is preserved; and (3) SBS with jejuno–ileal anastomosis (type 3), characterized by a remnant of at least 10 cm of terminal ileum in continuity with an intact colon.

### 2.5. Ethical Considerations

This study was approved by the Ethics Committee of our university hospital (R06-083). We used anonymized data extracted from medical records to ensure patient confidentiality throughout the study. Informed consent was obtained using the opt-out method due to the nature of the study. Because the participants were children, their parents or legal guardians were provided with the opportunity to opt-out on their behalf.

## 3. Results

This study consisted of two boys and two girls, aged between 11 and 60 months. The underlying conditions are shown in Table 1.

Case 1 was a girl with multiple intestinal atresia requiring resection of the ileocecal region. At the time of enterostomy closure on day 54, the remnant bowel length was 25 cm. At 7 months of age, she underwent serial transverse enteroplasty (STEP), which increased the bowel length to 47 cm (SBS type 2).

Case 2 involved a girl with an ileal perforation requiring resection of the ileocecal region. The patient underwent enterostomy closure at 8 months of age (SBS type 2). The remnant small intestine consisted of 60 cm of jejunum.

Case 3 involved a boy with an internal hernia that required ileal resection, with preservation of the proximal jejunum and terminal ileum. The enterostomy was closed at 3 months of age (SBS type 3). The remnant small intestine was 30 cm of jejunum and 5 cm of ileum in length.

Case 4 had jejunal volvulus caused by an intestinal duplication that required resection of the jejunum, resulting in the preservation of the ileum. The enterostomy closed at 1 month of age (SBS type 3). The remnant small intestine consisted only of the ileum, measuring 80 cm in length.

Changes in plasma citrulline levels and PN energy intake are summarized in Table 2.

Compared with baseline, plasma citrulline levels increased in all patients after 12 months of daily teduglutide treatment. In type 2 SBS (cases 1 and 2), the rates of plasma citrulline increase were 114% and 52%, respectively. The corresponding reductions in PN energy intake were 100% (case 1 with complete PN weaning) and 30% (case 2). In contrast, among type 3 SBS patients, case 3 showed a plasma citrulline increase rate of 13.6% and a PN reduction rate of 33%, while case 4 had a citrulline increase rate of 34% and a PN reduction rate of 73%. No cases of treatment discontinuation or teduglutide-related complications were observed.

## 4. Discussion

In this case series of four pediatric patients with SBS, all demonstrated increased plasma citrulline levels and reduced PN requirements after 12 months of daily teduglutide treatment. However, the degree of change in citrulline levels and PN requirements varied among cases. Cases 1 and 2, both categorized as SBS Type 2, showed substantial increases in citrulline and clear reductions in PN dependence. Case 3, classified as SBS Type 3 and retaining both jejunum and ileum, exhibited the highest relative increase in citrulline (136%); however, the absolute citrulline levels remained low, and the reduction in PN requirement was modest. Interestingly, Case 4, in which a long segment of ileum was preserved following jejunal resection, showed only a slight increase in citrulline relative to the bowel length, whereas the reduction in PN requirement was substantial.

Teduglutide treatment has been reported to increase plasma citrulline levels compared to both placebo and baseline values [4,13]; however, the degree of increase may vary depending on the anatomical configuration of the remnant bowel. Endogenous GLP-2 secretion from L cells is mainly produced in the ileum and colon [14]. GLP-2 receptor mRNA expression is the highest in the jejunum, followed by the ileum and colon [15]. Plasma citrulline mainly originates in the proximal small bowel [16]. Therefore, in patients with substantial preservation of the jejunum, endogenous GLP-2 secretion is presumed to be low, but GLP-2 receptor expression secretion is presumed to be high. In such patients, the administration of a GLP-2 analogue may result in a greater increase in plasma citrulline levels. In contrast, in patients with limited jejunal length but preservation of the ileum and colon, where endogenous GLP-2 secretion is relatively maintained and intestinal adaptation tends to occur, the likelihood of achieving parenteral support independence may be higher; however, the increase in plasma citrulline levels following teduglutide treatment may be less pronounced. Jeppesen reported that the largest absolute and percentage increases in plasma citrulline were observed in patients with a jejunostomy or ileostomy rather than in those with colon-in-continuity with ≥50% of colon remaining or <50% of colon remaining with colostomy [4]. In addition, patients with a jejunostomy or ileostomy were the only bowel anatomy subgroup that showed a significant decrease in parenteral support volume from baseline at week 24, compared to the placebo. The authors suggested that this might be because patients undergoing jejunostomy or ileostomy have larger target organs, with greater numbers of GLP-2 receptors and less endogenous GLP-2 secretion.

This study included one patient who underwent STEP. In a pig model of short bowel syndrome with 90% bowel resection, Chang demonstrated that serum citrulline levels were significantly higher in the STEP group than in the control group [17]. However, whether bowel segments that have undergone a STEP procedure respond to GLP-2 analogue treatment in the same manner as native, non-lengthened bowels has not yet been established. Therefore, whether residual bowel length should be evaluated based on pre- or post-STEP measurements when assessing plasma citrulline levels following teduglutide treatment remains unclear. Further accumulation of clinical cases is required to clarify these issues.

Regarding the study limitations, this case series had a small sample size; however, pediatric SBS is a rare condition, and patients often have heterogeneous clinical backgrounds. Additionally, this study lacked a valid control group for comparison, which restricts the ability to attribute the observed outcomes solely to teduglutide treatment. Several large cohort studies have shown that many infants with SBS can achieve enteral autonomy through natural intestinal adaptation alone, without the use of pharmacological agents such as GLP-2 analogues. For example, a Nordic multicenter study reported that 76% of pediatric patients with SBS were successfully weaned from PN support by a median age of 4.4 years [18]. Similarly, Sandy et al. found that 62% of infants achieved enteral autonomy within an average of 2.3 years, with residual bowel anatomy, including colon length and the presence of the ileocecal valve, identified as important predictors [19]. In the study by Proli et al. involving 55 infants with neonatal short bowel syndrome, 41% of patients classified as type 2 and 55% of those classified as type 3 were weaned off PN, with a median time to PN weaning of 2.14 years (IQR: 1.83–3.09) and 1.7 years (IQR: 1.34–2.16), respectively. Plasma citrulline levels significantly increased over time in patients who achieved enteral autonomy, while those who remained PN-dependent did not show a consistent increase in citrulline levels [7]. These data highlight the variability in natural adaptation and underscore the need to consider individual anatomical and developmental factors when evaluating the effects of teduglutide. Furthermore, the approach to reducing PN volume varies from case to case as each child differs in developmental stage and oral intake ability, making it challenging to apply a standardized protocol. Therefore, further detailed case reports, including anatomical information on the remnant bowel, are essential to better understand the clinical course and response to GLP-2 analogue treatment.

## Figures and Tables

**Table 1:** Patient characteristics and remnant intestinal features.

Case	Age (Months)	Sex	Primary Disease	SBS Type	Remnant Intestine	Residual Bowel Length (cm)	Ileocecal Valve
1	60	F	multiple intestinal atresias	2	jejunum	25→47 (STEP)	(−)
2	21	F	ileal perforation	2	jejunum	60	(−)
3	59	M	internal hernia	3	jejunum ileum	30 5	(+)
4	11	M	jejunal volvulus caused by intestinal duplication	3	ileum	80	(+)

**Table 2:** Changes in body weight, transthyretin, plasma citrulline levels, and parenteral nutrition requirements after 12 months of teduglutide treatment.

Case	Body Weight (kg)		Transthyretin (mg/dL)		Plasm Citrulline Level (nmol/L)			PN (kcal/kg/day)		
	Baseline	1 Year	Baseline	1 Year	Baseline	1 Year	Increase Rate	Baseline	1 Year	Reduction Rate
1	15.3 (−0.4SD)	17.2 (−0.4SD)	20	27.5	22.5	48.2	114%	15.9	0	−100%
2	8.8 (−1.6SD)	14.9 (1.5SD)	21.8	25.2	25.4	38.6	52%	50	35	−30%
3	16.0 (−0.6SD)	17.7 (−0.7SD)	19.4	21.8	11.7	27.6	136%	28.5	19.2	−33%
4	5.1 (−4.6SD)	10.9 (−0.6SD)	13.7	19.9	26.9	36.1	34%	48	13	−73%

## Data Availability

Data available on request due to restrictions.

## Abbreviations

The following abbreviations are used in this manuscript:

GLP-2	Glucagon-like peptide-2
PN	Parenteral nutrition
SBS	Short bowel syndrome
STEP	Serial transverse enteroplasty
